# Thyroid hormone, cortisol, interleukin-2, and procalcitonin regulate postoperative delirium in acute type A aortic dissection patients

**DOI:** 10.1186/s12872-022-02962-6

**Published:** 2022-11-24

**Authors:** Guo-Zhong Zheng, Xing-Feng Chen, Liang-Wan Chen, Zeng-Rong Luo

**Affiliations:** 1 Department of Cardiovascular Surgery and Cardiac Disease Center, Union Hospital, Fujian Medical University, Fuzhou, 350001 People’s Republic of China; 2 Key Laboratory of Cardio-Thoracic Surgery (Fujian Medical University), Fujian Province University, Fuzhou, People’s Republic of China

**Keywords:** Thyroid hormones, Cortisol, Postoperative delirium, IL-2, Aortic dissection

## Abstract

**Background:**

We assessed the relationships between levels of preoperative thyroid hormone (TH), cortisol, interleukin-2 (IL-2), and procalcitonin (PCT) and postoperative delirium (POD) in acute type A aortic dissection (ATAAD) patients receiving modified triple-branched stent-graft (MTBSG) implant surgeries.

**Methods:**

ATAAD patients received MTBSG implant surgeries in our hospital between February 2019 and December 2020 were recruited. We separated them into a POD and non-POD cohort and employed univariable and multivariable regression analysis to establish independent correlations between preoperative THs, cortisol, IL-2, and PCT and POD. In addition, we conducted stratification analyses to examine the link between pre-surgical THs and POD in normal TSH and lower TSH subgroups.

**Results:**

POD occurred in 78 of 224 patients (34.8%). POD patients exhibited markedly reduced preoperative free triiodothyronine (FT3) (*P* = 0.008) and free thyroxine (FT4) (*P* = 0.023) levels, while remarkably enhanced preoperative cortisol (*P* < 0.001), IL-2 (*P* < 0.001), and PCT (*P* < 0.001) levels. Based on multivariate regression analysis, reduced preoperative FT3 (*P* = 0.032), as well as augmented preoperative IL-2 (*P* = 0.001), cortisol (*P* < 0.001), and PCT (*P* = 0.016) were strong stand-alone risk factors for POD. Moreover, subgroup analysis found the association between FT3 (*P* = 0.029), FT4 (*P* = 0.042) and POD was both significant in patients with normal TSH levels.

**Conclusions:**

Reduced preoperative FT3 and elevated preoperative cortisol, IL-2, and PCT were strong indicators of POD in ATAAD patients. Hence, we recommend that the thyroid function, cortisol, PCT, and IL-2 should be evaluated prior to surgery in ATAAD patients.

## Introduction

Acute type A aortic dissection, also known as ATAAD, is a serious condition that has a high associated death rate. It requires immediate surgery [1], and postoperative delirium (POD) is a prominent postoperative complication [2]. POD is characterized by acute cognitive impairment, with fluctuating consciousness and attention following heart surgery [3]. Moreover, POD is intricately linked to reduced mobility, enhanced intensive care unit (ICU) stay, high costs, and undesirable patient prognosis [4].

Multiple reports suggested that some hormones and cytokines (e.g. thyroid hormones (THs) [5], cortisol [6], procalcitonin (PCT) [7], and interleukin-2 (IL-2) [8]) modulate the occurrence, progression, and physiology of POD.

Decrease of THs [5] and excessive release of glucocorticoid [9], owing to major surgery-related physiological and emotional stress, may accelerate POD occurrence. Moreover, a postoperative inflammatory response is also a known risk factor for POD development [10]. Following cardiac surgery with CPB, an inflammatory marker known as PCT may potentially serve as an effective early POD indication [7]. In addition, elevated IL-2 levels are strongly correlated with POD occurrence following coronary artery bypass grafting (CABG) surgery [8].

Thus far, the correlations between these hormones, cytokines, and POD have not been systematically investigated. Hence, here, we explored the potential links between pre-surgical hormones, cytokines, and POD in ATAAD patients, who underwent modified triple-branched stent-graft (MTBSG) implant surgeries.

## Methods and patients

### Patients selection

Our research received ethical approval from the Fujian Medical University Union Hospital (permission number: 2019KY019, date: 2019–01-31), and obtained informed consent from all participants prior to the initiation of the study.

We recruited ATAAD patients, who underwent MTBSG implant surgeries in the Fujian Union Hospital between February 2019 and December 2020. The following patients were included in our analysis: (1) over 18 years of age, and (2) provided informed consent. The following patients were eliminated from our analysis: (1) history of psychiatric or neurological diseases like depression, schizophrenia, stroke, and dementia (2) diagnosed with uremia, and liver cirrhosis (3) a lack of blood flow to the brain before surgery, (4) Shock caused by cardiac tamponade or hemodynamic instability before surgery, (5) liver enzymes over four folds that of baseline, (6) visual and/or hearing dysfunction, (7) post-surgical coma or death within 24 h after surgery, (8) underwent treatment with extracorporeal membrane oxygenation, (9) history of delirium, endocrine, and rheumatic immune disease, pituitary diseases, or consuming thyroid function- and cortisol content-regulating drugs.

### Anesthesia and surgery

Anesthesia (sevoflurane, midazolam, sufentanil, rocuronium, and dexmedetomidine) was provided both intravenously and via inhalation to all patients, and the dosage was based on patient weight. We also measured the nasopharyngeal and rectal temperatures, as well as the upper and lower limb blood pressure. Bispectrality Index (BIS) Monitoring System measured anesthetic depth, while Regional Oximetry System (VISTA, Covidien) monitored regional cerebral oxygen saturation (VISTA, Covidien). Low-flow CPB and cardiocirculatory arrest under profound hypothermia were used during surgery on the patients. For unilateral selective cerebral perfusion, the right axillary artery was used, whereas, for bilateral cerebral perfusion, both the right axillary artery and the left common carotid artery were used. The subsequent procedures are described in our prior publication [11]. Additionally, propofol was used to keep patients sedated after surgery in the intensive care unit (ICU). Moreover, if the patients were hemodynamically stable, with appropriate oxygenation and no blood loss, then they were also extubated.

### POD assessment

POD was assessed two times a day in the ICU or general ward three days after the operation. POD was evaluated in two stages: the Richmond agitation-sedation score (RASS) evaluated the consciousness level [12]. RASS values less than -4 were discarded. If the patient remained unconscious after 30 min, the sedative prescription was modified and the RASS was examined again. Scores above -4 indicated passage to the next step of the examination, which included the use of the ICU confusion assessment method (CAM-ICU) [13]. Upon alteration of the patient’s state of consciousness, the evaluation was completed instantly.

POD was described as a sudden start and variation in mental state as well as inattention and disordered thinking.

### Data collection

Patient characteristics, namely body mass index (BMI), age, sex, and comorbidities (diabetes, hypertension, drinking, and smoking history) were recorded for all patients. Also, measured was clinical information including pre-surgical examination of TSH, FT3, FT4, cortisol, IL-2, PCT, red blood cell (RBC), white blood cell (WBC), platelet, serum creatinine, hemoglobin, serum total bilirubin, serum albumin, serum globulin, D-Dimmer, and ejection fraction. The recorded intraoperative information included deep hypothermic circulatory arrest duration, aortic cross-clamp duration, cardiopulmonary bypass duration, lowest rectal temperature, as well as RBC and platelet transfusions. The postsurgical data included ICU retention period and in-hospital death.

### TH sampling

We analyzed the thyroid activities of all participants prior to surgery. Upon blood sample collection, the samples were instantly centrifuged and assessed. We evaluated the FT3, FT4, and TSH levels using corresponding thyroid functional kits (Beckman counter corporation, chemiluminescent immunoassay). We also obtained the peripheral blood samples prior to surgery to determine serum cortisol levels. These samples were immediately centrifuged and maintained in –80 °C until further analysis. An iodine cortisol radioimmunoassay kit was employed for serum cortisol monitoring, following kit protocols.

Furthermore, we also routinely monitored IL-2 and PCT contents a day prior to surgery (baseline), as well as on day 0 (upon ICU admission), 1, 2, and 3 post operation. All analyses were conducted in the hospital laboratory via a chemiluminescent microparticle immunoassay (PCT: ARCHITECT B R A H M S PCT, Abbott, Chicago, IL, USA; IL-2: IMMULITE® 1000 Immunoassay System (Siemens Healthcare Diagnostics GmbH, Munich, Germany).

### Definitions and end point

ATAAD was classified as a dissection affecting the ascending aorta with a < 14-day symptom onset [1]. Our major endpoint was POD.

### Statistical analysis

The Shapiro–Wilk test was employed for data distribution assessment. Continuous data are provided as means ± standard deviations. Inter-group analyses were carried out by t-tests for normally distributed data. Data with non-normal distribution are provided as medians and percentiles (interquartile range between the 25th and 75th percentiles) and analyzed via the non-parametric Mann–Whitney U test. A Friedman’s ANOVA with post-hoc tests was employed for the analysis of IL-2 and PCT within groups. The chi-squared and Fisher's exact tests were used to assess categorical data.

Multivariate regression was employed to identify stand-alone risk factors for POD. We also included unadjusted and fully adjusted analyses in this study. The confounders were selected depending on their associations with patient outcomes or effect estimates less than 0.05. To verify whether the effects of the levels of the THs on POD differed across various subgroups classified by TSH level, we also performed stratification analyses to establish differences in THs within various TSH-stratified subgroups. In subgroup comparisons, a TSH threshold was employed [14], and two stratifications were described as follows: TSH ≤ 0.49 mIU/L was the reduced TSH cohort, and TSH > 0.49 mIU/L was the normal TSH cohort. The cutoff for statistical significance was determined to be a two-tailed P value < 0.05. The software packages R (R-project.org, The R Foundation) and SPSS 26.0 were used throughout the conducted analysis.

## Results

### Baseline features between POD and non-POD patients

**Fig. 1:**
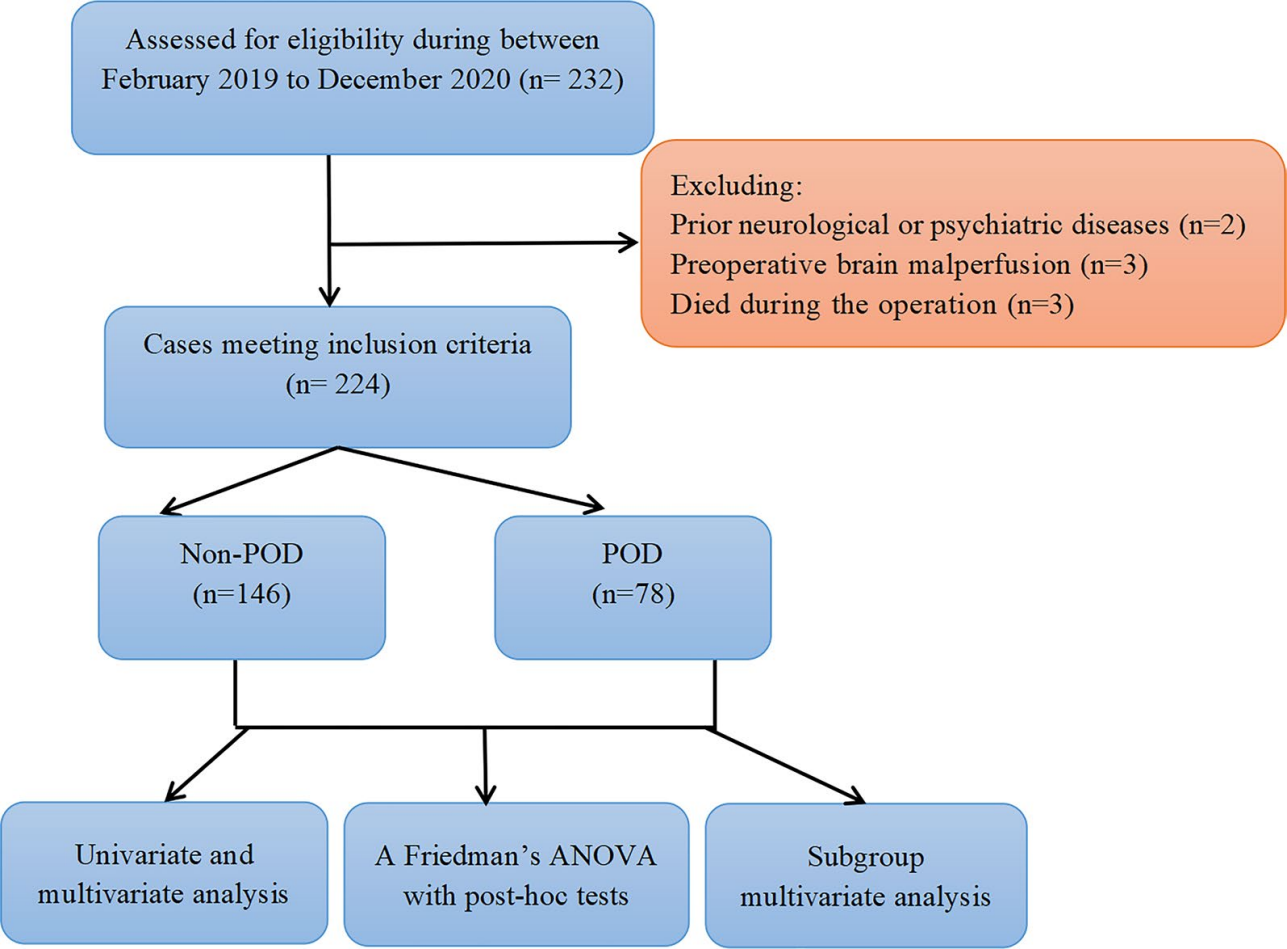
Flow chart of the study. (POD, postoperative delirium)

Overall, 232 patients received MTBSG surgery during our investigative period. Upon exclusion of 8 patients (2 patients with prior neurological diseases, 3 patients with preoperative brain malperfusion and 3 patients expired during surgery), 224 patients were selected for the final analysis. Among them, 78 patients (34.8%) experienced POD after MTBSG surgery. Figure 1 presents a summary of our study design.

The POD patient group displayed enhanced BMI (*P* = 0.007), advanced age (*P* = 0.007), more alcohol intake (*P* = 0.021), and elevated serum total bilirubin levels (*P* = 0.002). In addition, relative to non-POD patients, POD patients exhibited markedly reduced pre-surgical free triiodothyronine (FT3) (4.57 ± 1.05 vs. 5.08 ± 1.78 pmol/L, *P* = 0.008), free thyroxine (FT4) (11.69 ± 6.40 vs. 13.30 ± 4.08 pmol/L, *P* = 0.023), and significantly elevated pre-surgical cortisol (399.31 (334.95,451.85) vs. 316.64 (281.18,376.56) nmol/L, *P* < 0.001), IL-2 (3.21 (2.72,3.69) vs. 2.56 (1.94,3.16) pg/ml, *P* < 0.001), and PCT levels (0.05 (0.04,0.06) vs. 0.03 (0.02,0.05) ng/mL, *P* < 0.001) (Table 1).

**Table 1:** Baseline characteristics

Variables	Without POD n = 146	With POD n = 78	χ^2^ /Z/t	*P* value
*Demographics*				
Age (years), mean ± SD	51.20 ± 11.38	55.58 ± 11.42	2.741	**0.007**^**a**^
BMI (kg/m^2^), mean ± SD	24.05 ± 3.69	25.63 ± 4.35	2.726	**0.007**^**a**^
Sex (Female), n (%)	36 (24.7%)	20 (25.6%)	0.026	1.000^b^
*Comorbidities*				
Hypertension, n (%)	108 (74.0%)	56 (71.8%)	0.123	0.726 ^b^
Diabetes, n (%)	16 (11.0%)	5 (6.4%)	1.238	0.266^b^
Smoking, n (%)	33 (22.6%)	25 (32.1%)	2.365	0.124^b^
Drinking history, n (%)	30 (20.5%)	27 (34.6%)	5.303	**0.021**^**b**^
*Laboratory values*				
Preoperative TSH (mIU/L), mean ± SD	2.20 ± 1.75	2.02 ± 1.65	0.748	0.455^a^
Preoperative FT3 (pmol/L), mean ± SD	5.08 ± 1.78	4.57 ± 1.05	2.694	**0.008 **^**a**^
Preoperative FT4 (pmol/L), mean ± SD	13.30 ± 4.08	11.69 ± 6.40	2.291	**0.023 **^**a**^
Preoperative cortisol (nmol/l), median (Q1, Q3)	316.64 (281.18,376.56)	399.31 (334.95,451.85)	7.005	**< 0.001**^**c**^
Preoperative IL-2 (pg/ml), median (Q1, Q3)	2.56 (1.94,3.16)	3.21 (2.72,3.69)	6.201	**< 0.001**^**c**^
Preoperative PCT (ng/mL), median (Q1, Q3)	0.03 (0.02,0.05)	0.05 (0.04,0.06)	6.306	**< 0.001**^**c**^
White blood cell count (× 10^9^/L), median (Q1, Q3)	9.0 (7.2, 12.2)	9.2 (7.8, 13.4)	1.440	0.120 ^c^
Red blood cell count (× 10^12^/L), median (Q1, Q3)	5.2 (4.0, 5.8)	5.1 (4.0, 5.5)	0.336	0.870 ^c^
Hemoglobin (g/L), median (Q1, Q3)	120 (112, 140)	118 (112, 142)	0.205	0.770 ^c^
Platelet count (× 10^9^/L), median (Q1, Q3)	170 (135, 222)	160 (134, 199)	1.995	0.354 ^c^
Serum creatinine (μmol/L), median (Q1, Q3)	80.7 (75.0, 119.4)	96.7 (80.0, 120.0)	1.800	0.341 ^c^
Serum total bilirubin (umol/L), median (Q1, Q3)	48.8 (27.5, 74.5)	76.9 (65.0, 98.8)	4.780	**0.002** ^c^
Serum albumin (g/L), median (Q1, Q3)	37.4 (35.5, 42.0)	38.1 (35.0, 42.2)	0.259	0.805 ^c^
Serum globulin (g/L), median (Q1, Q3)	24.0 (22.2, 28.0)	24.3 (22.3, 28.5)	0.411	0.693 ^c^
D-Dimmer (ug/L), median (Q1, Q3)	183 (45,355)	194 (58,349)	0.570	0.580 ^c^
Ejection fraction (%), median (Q1, Q3)	61 (54, 66)	60 (53, 66)	0.300	0.799 ^c^
*Intraoperative data*				
Catogeries of surgery, n (%)			3.064	0.559 ^b^
Isolated AAD surgery	97 (66.4)	47 (60.3)		
Combined valve surgery	30 (20.5)	15 (19.2)		
Combined coronary artery bypass grafting	9 (6.2)	6 (7.7)		
Combined valve and coronary surgery	8 (5.5)	7 (9.0)		
Combined other types of cardiac surgery	2 (1.4)	3 (3.8)		
Deep hypothermic circulatory arrest time, min, median (Q1; Q3)	16.5 (13.5, 20.5)	17.5 (14.5, 22.5)	1.032	0.505 ^c^
Cardiopulmonary bypass time, min, median (Q1; Q3)	145 (100, 186)	155 (115, 190)	0.575	0.668^c^
Aortic cross clamp time, min, median (Q1; Q3)	59.4 (48.5, 70.0)	62.3 (52.8, 72.6)	1.008	0.588^c^
Lowest rectal temperature (°C), mean ± SD	23.30 ± 2.45	23.55 ± 2.60	0.712	0.477^a^
Transfusion of red blood cells (units), mean ± SD	4.50 ± 3.50	4.85 ± 3.80	0.692	0.490 ^a^
Transfusion of platelet (units), mean ± SD	8.88 ± 3.50	9.40 ± 3.50	1.059	0.291 ^a^

The Shapiro–Wilk test was used to assess data distributions. Results are expressed as n (%) or mean ± standard deviation (SD), median (Q1; Q3)

*POD* postoperative delirium, *BMI* body mass index, *Q1* first quartile, *Q3* third quartile, *FT3* free triiodothyronine, *FT4* free thyroxine, *TSH* thyroid stimulating hormone, *IL-2* interleukin-2, *PCT* procalcitonin, *AAD* acute aortic dissection

^a^Student’s t test

^b^chi-squared or Fisher’s exact test. ^c^ Wilcoxon rank-sum test

Bold mean statistical significances were determined due to a two-tailed *P* value < 0.05

### Intraoperative patient profile

Table 1 summarizes the intraoperative profiles of both patient cohorts. The catogeries of surgery, DHCAD, CPBD, ACCD, lowest rectal temperature, RBC transfusion, and platelet transfusion showed no difference between the POD and non-POD cohorts (all *P* > 0.05).

### Multivariate analyses

Following adjustments for age, sex, BMI, drinking history, total serum bilirubin, platelet, hemoglobin, serum creatinine, intraoperative packed RBC transfusion, deep hypothermic circulatory arrest time, cardiopulmonary bypass time and aortic cross clamp time, our multivariate regression analyses revealed that significantly low levels of preoperative FT3 (95% CI, OR = 0.880, 0.665–0.941, *P* = 0.032), and considerably high levels of preoperative cortisol (95% CI, OR = 1.019, 1.013–1.025, *P* < 0.001), IL-2 (95% CI, OR = 2.497, 1.478–4.218, *P* = 0.001), and PCT (95% CI, OR = 7.768, 1.471–41.022, *P* = 0.016) were independently related to POD development (Table 2).

**Table 2:** Univariate and multivariate logistic regression analysis of the related significant hormones and inflammatory predictors of POD in AAD patients

Variables	Univariate analysis		Multivariate analysis	
	OR(95%CI)	*P*-value	OR(95%CI)	*P*-value
Preoperative FT3 (pmol/L)	0.672 (0.482, 0.802)	0.008	0.880 (0.665, 0.941)	**0.032**
Preoperative FT4 (pmol/L)	0.856 (0.539, 0.961)	0.024	0.877 (0.601, 1.312)	0.240
Preoperative cortisol (nmol/l)	1.018 (1.013,1.024)	< 0.001	1.019 (1.013,1.025)	**< 0.001**
Preoperative IL-2 (pg/ml)	2.351 (1.510,3.660)	< 0.001	2.497 (1.478,4.218)	**0.001**
Preoperative PCT (ng/mL)	6.544 (1.551,27.605)	0.011	7.768 (1.471,41.022)	**0.016**

Univariate analysis adjusted for: None

Multivariate analysis adjusted for: age; sex; body mass index; drinking history; serum total bilirubin; platelet; hemoglobin; creatinine; intraoperative transfusion of packed red blood cell; deep hypothermic circulatory arrest time; cardiopulmonary bypass time; aortic cross clamp time

Bold mean statistical significances were determined due to a two-tailed *P* value < 0.05

*POD* postoperative delirium, *FT3* free triiodothyronine, *FT4* free thyroxine, *TSH* thyroid stimulating hormone, *IL-2* interleukin-2, *PCT* procalcitoninis, *AAD* acute aortic dissection, *OR* odds ratio, *CI* confidence interval

### Changes in IL-2 and PCT concentrations over time

**Fig. 2:**
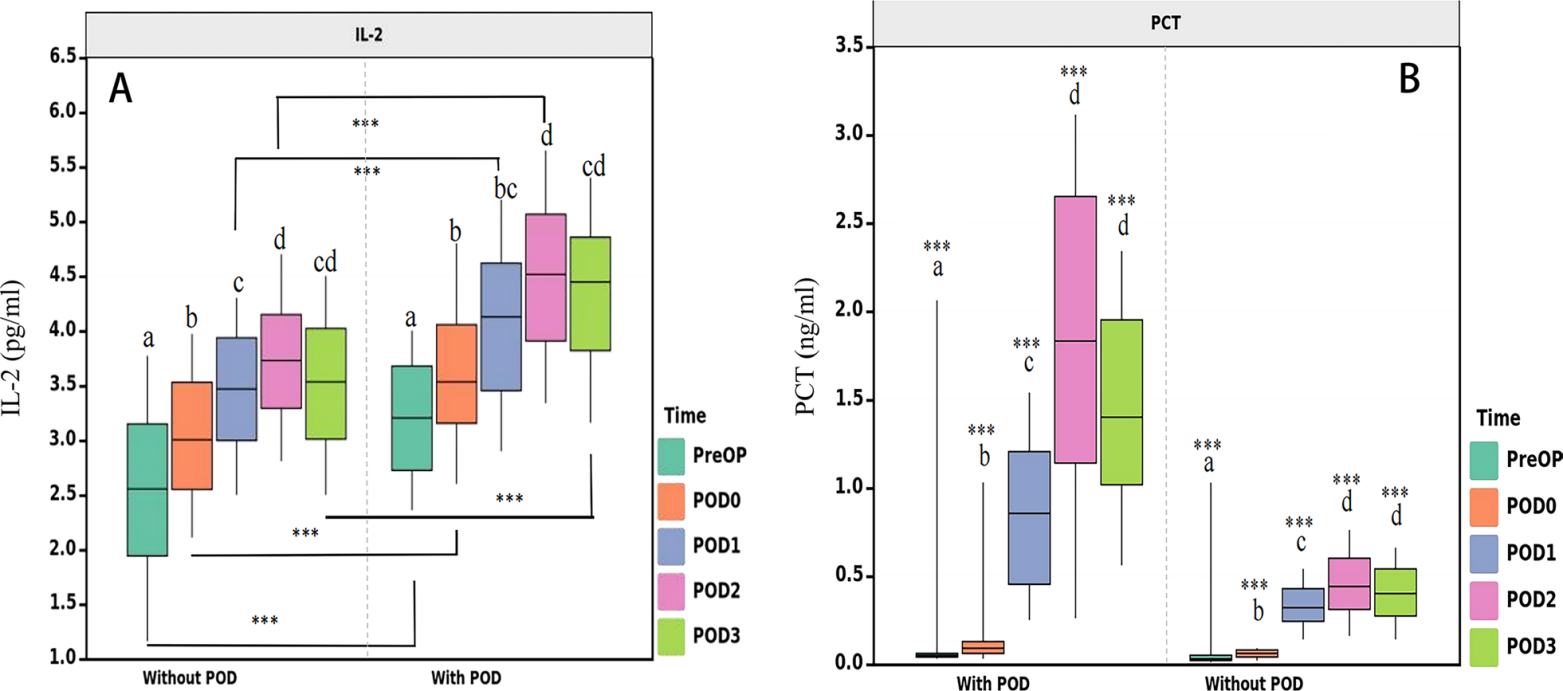
Daily comparison of the IL-2 and PCT levels between the POD and non-POD cohorts. (“abcd” represents the comparison at different time point by letter notation method (intra-group comparison); “***”indicates a significant difference (*P* < 0.001) between the POD and non-POD cohorts at each time point (inter-group comparison); with POD, patients with postoperative delirium; without POD, patients without postoperative delirium; PreOP, preoperative; POD0, upon admission to the intensive care unit; POD1, postoperative day 1; POD2, postoperative day 2; POD3, postoperative day 3; IL-2, interleukin-2; PCT, procalcitonin)

Figure 2 presents a daily comparison of the IL-2 and PCT levels between the POD and non-POD cohorts.

### IL-2

In the POD cohort, the median pre-surgical IL-2 levels were 3.21 pg/ml (IQR 2.72–3.69), whereas, in the non-POD group, the median pre-surgical IL-2 levels were 2.56 ng/mL (IQR 1.94–3.16) (*P* < 0.001). Following the operation, the IL-2 levels were markedly elevated in the POD versus non-POD cohort upon admission to the ICU (day 0: 3.54 ng/mL, IQR 3.13–4.08 vs. 3.01 ng/mL, IQR 2.54–3.54, *P* < 0.001), and on the subsequent days (day 1: 4.13 ng/mL, IQR 3.43–4.64 vs. 3.47 ng/mL, IQR 2.98–3.95, *P* < 0.001; day 2: 4.52 ng/mL, IQR 3.88–5.07 vs. 3.73 ng/mL, IQR 3.29–4.16, *P* < 0.001; day 3: 4.45 ng/mL, IQR 3.77–4.87 vs. 3.54 ng/mL, IQR 3.00–4.03, *P* < 0.001, respectively, in the POD and non-POD cohorts), and it demonstrated a peak value on post-surgical day 2. An Friedman’s ANOVA test analysis of the alterations in IL-2 content within each cohort revealed marked alterations in the POD (*P* < 0.001) and non-POD cohorts (*P* < 0.001). Based on our post-hoc evaluation, a marked rise was observed in the IL-2 levels on days 0, 1, 2, and 3, relative to baseline in both cohorts; and a marked rise was observed on days 0, 1, and 2 in the group without POD and days 0 and 2 in the group with POD compared to the previous day's level (Fig. 2A).

### PCT

There was a significant difference between the pre-surgery PCT values in the POD and non-POD groups, with medians of 0.05 ng/ml (IQR 0.04–0.06) and 0.03 ng/ml (IQR 0.02–0.03), respectively (*P* < 0.001). Following surgery, the PCT concentration was markedly elevated in the POD versus non-POD cohort upon ICU admission (day 0: 0.09 ng/ml, IQR 0.06–0.13 vs. 0.06 ng/ml, IQR 0.04–0.08, *P* < 0.001), and on the subsequent days (day 1: 0.86 ng/ml, IQR 0.44–1.22 vs. 0.43 ng/ml, IQR 0.24–0.54, *P* < 0.001; day 2: 1.83 ng/ml, IQR 1.10–2.66 vs. 0.44 ng/ml, IQR 0.31–0.60, *P* < 0.001; day 3: 1.40 ng/ml, IQR 1.01–1.97 vs. 0.40 ng/ml, IQR 0.27–0.54, *P* < 0.001, respectively, in the POD and non-POD cohorts), and the peak value was reached on the second day post operation. Based on our analysis of altering PCT concentrations over time within each cohort, we observed marked alterations in the POD (*P* < 0.001, Friedman’s ANOVA test) versus non-POD (*P* < 0.001) cohort. Our post-hoc evaluation revealed a marked rise in PCT levels on days days 0, 1, 2, and 3, relative to baseline in both cohorts; and a marked rise was observed on days 0, 1, and 2 in both cohorts compared to the previous day's level (Fig. 2B).

### Subgroup analyses

The TSH concentration was used to classify subgroup analyses (Table 3). In patients with normal TSH concentrations, we discovered a significant relation between FT3 (OR = 0.772 95% CI, 0.583–0.910, *P* = 0.029), FT4 (OR = 0.908 95% CI, 0.715–0.988, *P* = 0.042) and POD; but in patients with lower TSH concentrations, we failed to found this relation (*P* = 0.556 and *P* = 0.663, respectively).

**Table 3:** Subgroup analysis of the associations between POD and the thyroid hormone

Variables	Univariate analysis			Multivariate analysis	
	OR(95%CI)		*P*-value	OR(95%CI)	*P*-value
*FT3 (pmol/L)*					
Normal TSH	0.557 (0.350, 0.743)	0.05	0.023	0.772 (0.583, 0.910)	**0.029**
Lower TSH	1.336 (0.538, 4.020)	0.31	0.386	0.850 (0.684, 1.559)	0.556
FT4 (pmol/L)					
Normal TSH	0.624 (0.447, 0.800)	0.99	0.028	0.908 (0.715, 0.988)	**0.042**
Lower TSH	0.758 (0.578, 1.369)	0.65	0.545	0.754 (0.522, 1.982)	0.663

Bold mean statistical significances were determined due to a two-tailed *P* value < 0.05

## Discussion

Emerging evidences suggest that POD is strongly correlated with multiple pre-surgical factors including age, alcohol addiction, electrolyte or glucose dysregulation, reduced functional and cognitive capacity, and type of surgery [15]. However, there are limited studies on the relationships between hormones/inflammatory cytokines and POD. In this study, we adjusted the pre- and intra-operative predictor factors, namely, sex, BMI, drinking history, serum total bilirubin, platelet, hemoglobin, creatinine, intraoperative packed RBC transfusion, deep hypothermic circulatory arrest time, cardiopulmonary bypass time, aortic cross clamp time, and identified the stand-alone POD risk factors for patients with ATAAD. Based on our analysis, reduced preoperative free FT3, and elevated cortisol, IL-2, and PCT levels are strongly associated with POD occurrence.

### TH

Patients diagnosed with clinical hypothyroidism and cerebral dysfunction exhibit reduced T3 levels and enhanced POD incidences [16]. The severity of thyroid functional impairment is directly associated with disease status, reduced TH content, and poor prognosis in POD patients [17]. POD occurs in about 37.86% of ATAAD patients after MTBSG implantation [2]. Moreover, POD patients exhibit reduced pre-surgical free FT3 levels, which corroborates our findings [18]. Non-thyroidal illness syndrome (NTIS) is characterized by TH dysregulation in individuals who do not have a thyroid-related illness, such as infectious infections, burns, cancer, or trauma [19]. NTIS brought on by critical illness is manifested by a marked reduction in serum thyroid-stimulating hormone (TSH), triiodothyronine (T3), and thyroxine (T4) [20]. This is also prevalent in other serious conditions, including acute myocardial infarction [21], heart failure [22], and post-cardiac surgery [23]. Under these circumstances, alterations in the thyroid activity negatively modulate patient prognosis, thus, resulting in POD [5]. In this study, we demonstrated marked associations between reduced FT3 concentration and POD in ATAAD patients. Acute inactivation of THs through inner-ring deiodination has been reported to be a compensatory mechanism that restores the body to baseline, lowers systemic energy consumption and metabolic rate, and improves survival [24]. However, dysregulated TH levels can potentially alter neurotransmitter synthesis and cytokine release, which eventually contributes to POD occurrence [25]. TH level aberrations can be partially diminished using cytokines or other inflammatory factors, which act upon the thyroid gland, hypothalamus, hepatic deiodinase system, pituitary gland, as well as the interaction between thyroxine and thyroid-associated globulin.

### Cortisol

This study also assessed the association between pre-surgical cortisol levels and POD in ATAAD patients, who underwent MTBSG surgery. Cortisol content is known to regulate cognitive dysfunction during POD after coronary bypass [9]. There are further evidences suggesting that the hypothalamic–pituitary–adrenal (HPA) axis disturbance, in combination with the enhanced release of inflammatory factors and cortisol, modulates cognitive decline and impairment [26]. Moreover, POD patients exhibit markedly elevated pre-surgical cerebrospinal fluid cortisol levels [27]. Likewise, high cortisol concentration is also prevalent in Alzheimer’s, dementia, and mild cognitive dysregulation patients [28]. These evidences suggest that the dysregulated HPA axis produces high levels of cortisol, which promote cognitive impairment, which results in POD [28]. The mechanism may involve that glucocorticoids inhibit endothelial cell proliferation and turnover in the hippocampus and prefrontal cortex [29], whilst HPA axis dysregulation results in decreased hippocampus volume [30]. In this investigation, we demonstrated a strong association between plasma cortisol concentration and POD. Acute damage to blood vessels and severe pain markedly enhance HPA axis activity, which, in turn, activates the immune system, thus elevating plasma cortisol and inflammatory cytokine levels, which ultimately contribute to the brain neuroendocrine and neurotransmitter dysbalance, which results in POD [31]. Thus, we measured cortisol prior to surgery to examine the validity of the connection between higher pre-surgical cortisol concentration and POD.

### IL-2

IL-2 is commonly used as a therapy for metastatic malignancy patients. More than half of patients who had CABG surgery were found to suffer from significant behavioral and/or cognitive impairment (disorientation and cognitive decline). Acute treatment was necessary for 34% of patients because of the behavioral changes [32]. IL-2 levels in the peripheral immune system and the brain were shown to be crucial for promoting either neurodegeneration or neuroregeneration [33] in animal experiments. We next examined the relationship between augmented IL-2 levels and POD among MTBSG implantation patients. AAD induces severe pain, which, in turn, activates complex neuroendocrine and tissue responses that protect the body from injury, and aid in the recovery of body integrity. Unfortunately, this also initiates a “hormonal storm” involving the sympathetic nervous system, sympatho-adrenomedullary, HPA activation, and periphery, as inflammatory factors like IL-2 are released at the injury site [34]. IL-2 is a T cell-released pro-inflammatory cytokine. Multiple sources suggest an active role of inflammatory molecules in POD pathogenesis [35]. Moreover, for both delirium and dementia, cognitive impairment is regulated by cytokines. For example, IL-2 promoted the survival and extension of cultured neurons, while encouraging oligodendrocyte maturation and proliferation, as well as a behavioral shift within the CNS [36].

### PCT

Another major finding of our investigation was that enhanced pre-surgical PCT is a stand-alone regulator of POD occurrence after MTBSG surgery, based on our multivariate logistic regression model. PCT is a precursor of the calcitonin peptide and is used as an inflammation biomarker. It is widely used in identifying bacterial infections and in monitoring antibiotic therapy in septic patients. Interestingly, increasing literature suggests that pre-surgical PCT can serve as a biomarker for adverse patient prognosis in non-infectious diseases. A prior investigation reported the significance of pre-surgical PCT in predicting POD following cardiac surgery [7]. Based on their observation, about 50% of cardiac surgery patients experienced POD, and only 27% of the non-POD patients exhibited pre-surgical PCT above the normal range. Furthermore, they revealed, based on multivariate logistic regression data, that the preoperative PCT markedly enhanced POD risk, with an odds ratio of 3.05. Receiving a cardiopulmonary bypass (CPB) activates the systemic inflammatory response, which, in turn, enhances the synthesis of a myriad of inflammatory factors both during and after surgery [37].

POD etiology is multifactorial. In this study, our multivariable model of POD prediction after MTBSG surgery included preoperative PCT levels, with an OR of 1.06, even after adjustments for confounding factors like age and CPB duration. Our results also correlated with prior published research [7]. In AAD patients, blood vessel injury activates the systemic inflammatory response. Subsequently, multiple inflammatory factors, including PCT, are secreted, which leads to systemic effects like vasodilatation and microcirculatory dysregulation [38]. Additional inflammatory factors are synthesized by microglial cells after they have been activated by components released during the systemic inflammation response via the blood–brain barrier (BBB), which is comparable to the POD neuroinflammatory hypothesis. As a result, the brain tissue, neuronal activity, the neurotransmitter system, and synaptic conduction become impaired, resulting in significant leakage from the intercellular connections of the BBB cells [39].

In this study, we evaluated PCT since it is easy to obtain routine PCT readings, and prior studies confirmed that PCT is predictive in identifying problems after cardiac surgery [40]. McGrane et al. previously showed that increased PCT levels upon ICU admission indicate long-term acute brain dysregulation in non-cardiac ICU patients. This shows that inflammation and POD 43 are closely linked [41]. We demonstrated markedly upregulated median PCT levels following surgery, with a peak on post-surgical day 2. Moreover, the PCT levels were markedly elevated in POD versus non-POD patients. Since PCT evaluations are easily available (point of care testing or hospital laboratory), and they are quick and cost-effective, they may be used as an additional early predictor of POD in ATAAD patients post MTBSG surgery.

### Limitations

Firstly, our cross-sectional study limits the establishment of a causal or temporal association between pre-surgical THs, cortisol levels, and POD in ATAAD patients. Secondly, since all patients received CTA, we were unable to exclude thyroid hormonal changes caused by iodinated contrast media, even though the prevalence of this is relatively low. In addition, here, we did not analyze the dynamic information involving the influence of THs and cortisol on POD. Lastly, we are aware that the peripheral recordings of these hormones and cytokines are indirect indexes of central neurotransmitter function, and that extrapolation to brain function is somewhat speculative.

## Conclusions

**Fig. 3:**
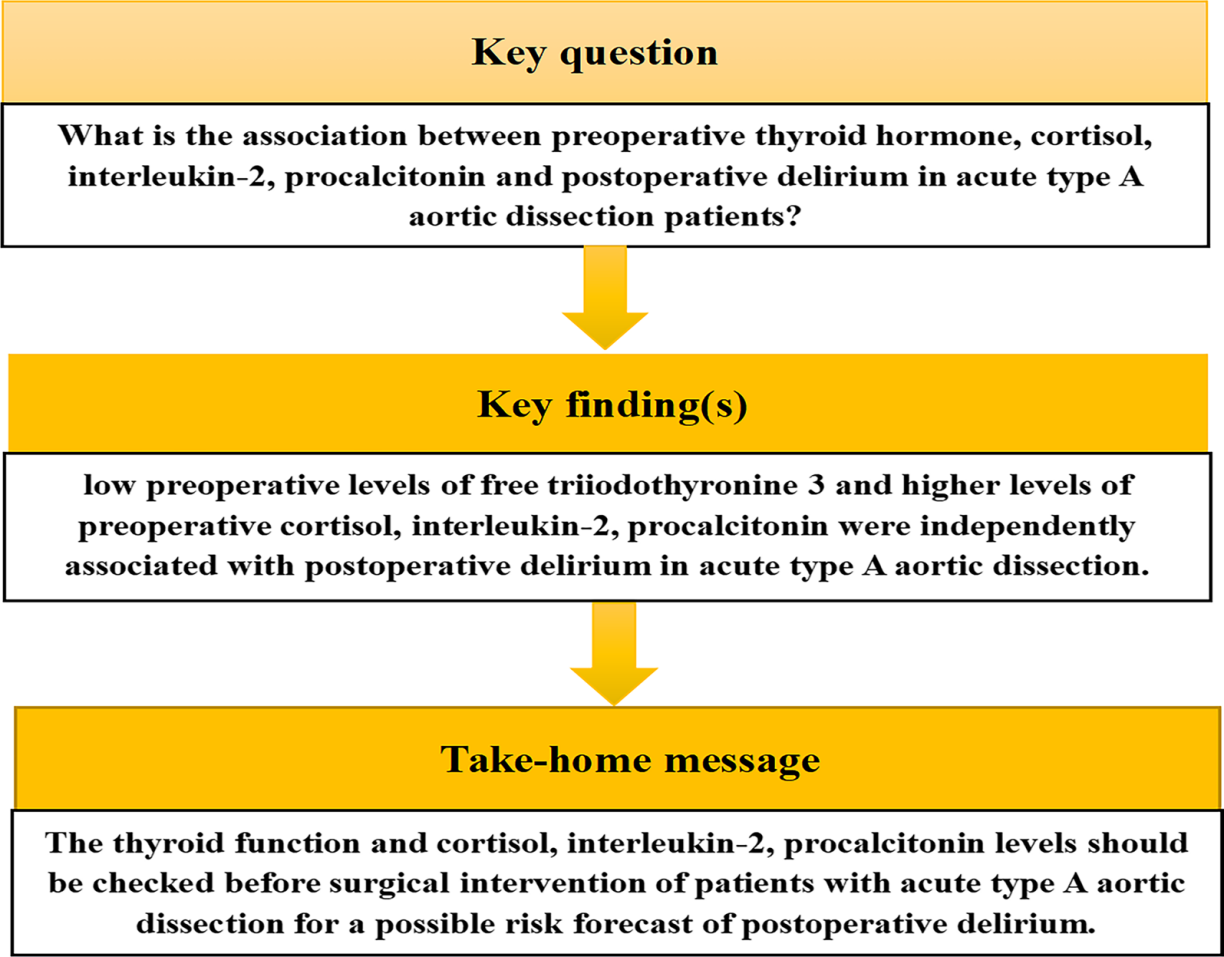
An illustrative summary of our findings

Figure 3 illustrates a summary of our findings. First, we demonstrated that reduced pre-surgical FT3, and elevated pre-surgical cortisol, IL-2, and PCT were stand-alone indicators of POD ATAAD patients. In addition, the overall influence of reduced FT3 and FT4 contents on POD was more prominent in the normal TSH patients. Lastly, the dynamic alterations in the IL-2 and PCT concentrations in POD patients were markedly elevated, compared to non-POD patients a few days post surgery. Given this evidence, we recommend close monitoring of the thyroid function, cortisol, IL-2, and PCT levels prior to surgery in ATAAD patients to better assess their risk of developing POD.

## Data Availability

The data that support the findings of this study are available from Fujian Cardiac Medical Center but restrictions apply to the availability of these data, which were used under license for the current study, and so are not publicly available. Data are however available from Zeng-Rong Luo author upon reasonable request and with permission of Fujian Cardiac Medical Center.

## Abbreviations

ATAAD: Acute type A aortic dissection
POD: Postoperative delirium
TH: Thyroid hormone
TSH: Thyroid-stimulating hormone
FT3: Free triiodothyronine
FT4: Free thyroxine
NTIS: Non-thyroidal illness syndrome
IL-2: Interleukin-2
PCT: Procalcitonin
CPB: Cardiopulmonary bypass
HPA: Hypothalamic–pituitary–adrenal
ICU: Intensive care unit
NYHA: New York Heart Association
RFs: Risk factors
MTBSG: Modified triple-branched stent-graft
CI: Confidence interval
OR: Odds ratio
Q1: First quartile
Q3: Third quartile

## References

[1] Raimund E, Victor A, Catherine B, Eduardo B, Di Roberto B, Holger E (2014). ESC Guidelines on the diagnosis and treatment of aortic diseases: document covering acute and chronic aortic diseases of the thoracic and abdominal aorta of the adult. The Task Force for the Diagnosis and Treatment of Aortic Diseases of the European Society of Cardiology (ESC). Eur Heart J.

[2] Lin Y, Chen Q, Zhang H, Chen L-W, Peng Y, Huang X (2020). Risk factors for postoperative delirium in patients with triple-branched stent graft implantation. J Cardiothorac Surg.

[3] Shadvar K, Baastani F, Mahmoodpoor A, Bilehjani E (2013). Evaluation of the prevalence and risk factors of delirium in cardiac surgery ICU. J Cardiovasc Thorac Res.

[4] Ralph FM, Vallire H, Sheri AD, Lucille T (2015). Outcomes associated with postoperative delirium after cardiac surgery. Am J Crit Care.

[5] Mafrica F, Fodale V (2008). Thyroid function, Alzheimer’s disease and postoperative cognitive dysfunction: a tale of dangerous liaisons?. J Alzheimers Dis.

[6] Eshmawey M, Arlt S, Ledschbor-Frahnert C, Guenther U, Popp J (2019). Preoperative depression and plasma cortisol levels as predictors of delirium after cardiac surgery. Dement Geriatr Cogn Disord.

[7] Kupiec A, Adamik B, Kozera N, Gozdzik W (2020). Elevated procalcitonin as a risk factor for postoperative delirium in the elderly after cardiac surgery-a prospective observational study. J Clin Med.

[8] Kazmierski J, Banys A, Latek J, Bourke J, Jaszewski R (2014). Raised IL-2 and TNF-α concentrations are associated with postoperative delirium in patients undergoing coronary-artery bypass graft surgery. Int Psychogeriatr.

[9] Kazmierski J, Kloszewska I (2011). Is cortisol the key to the pathogenesis of delirium after coronary artery bypass graft surgery?. Crit Care.

[10] José R (2018). Maldonado: delirium pathophysiology: an updated hypothesis of the etiology of acute brain failure. Int J Geriatr Psychiatry.

[11] Chen L-W, Dai X-F, Xi-Jie Wu, Liao D-S, Yun-Nan Hu, Zhang H (2017). Ascending aorta and Hemiarch replacement combined with modified triple-branched stent graft implantation for repair of acute DeBakey type I aortic dissection. Ann Thorac Surg.

[12] Sessler CN, Gosnell MS, Grap MJ, Brophy GM, O'Neal PV, Keane KA (2002). The Richmond agitation-sedation scale: validity and reliability in adult intensive care unit patients. Am J Respir Crit Care Med.

[13] Smulter N, Lingehall HC, Gustafson Y, Olofsson B, Engstrom KG (2015). Validation of the confusion assessment method in detecting postoperative delirium in cardiac surgery patients. Am J Crit Care.

[14] Pedro I, María AB, Rafael S, Juan JD (2017). Thyroid dysfunction and kidney disease: an update. Rev Endocr Metab Disord.

[15] Praticò C, Quattrone D, Lucanto T, Amato A, Penna O, Roscitano C (2005). Drugs of anesthesia acting on central cholinergic system may cause post-operative cognitive dysfunction and delirium. Med Hypotheses.

[16] van der Mast RC, van den Broek WW, Fekkes D, Pepplinkhuizen L, Habbema JD (2000). Is delirium after cardiac surgery related to plasma amino acids and physical condition?. J Neuropsychiatry Clin Neurosci Winter.

[17] El-Kaissi S, Mark AK, Michael B, Jack RW (2005). Acute delirium in the setting of primary hypothyroidism: the role of thyroid hormone replacement therapy. Thyroid.

[18] Lingzhi C, Hao Z, Weijian H, Gaoshu Z, Chengchao S, Changxi C (2016). Outcome predictors in patients presenting with acute aortic dissection. J Cardiothorac Vasc Anesth.

[19] Jabbar A, Pingitore A, Pearce SH, Zaman A, Iervasi G, Razvi S (2017). Thyroid hormones and cardiovascular disease. Nat Rev Cardiol.

[20] Senniappan S, Brown RE, Hussain K (2014). Sirolimus in severe hyperinsulinemic hypoglycemia. N Engl J Med.

[21] Li L, Guo CY, Yang J, Jia EZ, Zhu TB, Wang LS (2011). Negative association between free triiodothyronine level and international normalized ratio in euthyroid subjects with acute myocardial infarction. Acta Pharmacol Sin.

[22] Kannan L, Shaw PA, Morley MP, Brandimarto J, Fang JC, Sweitzer NK (2018). Thyroid dysfunction in heart failure and cardiovascular outcomes. Circ Heart Fail.

[23] Iervasi G, Pingitore A, Landi P, Raciti M, Ripoli A, Scarlattini M (2003). Low-T3 syndrome: a strong prognostic predictor of death in patients with heart disease. Circulation.

[24] Zaloga GP, Chernow B, Smallridge RC, Zajtchuk R, Hall-Boyer K, Hargraves R (1985). A longitudinal evaluation of thyroid function in critically ill surgical patients. Ann Surg.

[25] Tajana ZB, Dinko T, Ante S, Daniela B-P, Robert B, Marko B (2012). Pathophysiology of delirium. Acta Med Croatica.

[26] Kazmierski J, Banys A, Latek J, Bourke J, Jaszewski R, Sobow T (2014). Mild cognitive impairment with associated inflammatory and cortisol alterations as independent risk factor for postoperative delirium. Dement Geriatr Cogn Disord.

[27] Andrew P, de Annick V, Scott DM, Fiona G, Timothy OW, Ian RA (2010). Cerebrospinal fluid cortisol levels are higher in patients with delirium versus controls. BMC Res Notes.

[28] Ouanes S, Popp J (2019). High cortisol and the risk of dementia and Alzheimer’s disease: a review of the literature. Front Aging Neurosci.

[29] Ekstrand J, Hellsten J, Tingström A (2008). Environmental enrichment, exercise and corticosterone affect endothelial cell proliferation in adult rat hippocampus and prefrontal cortex. Neurosci Lett.

[30] Murray F, Smith DW, Hutson PH (2008). Chronic low dose corticosterone exposure decreased hippocampal cell proliferation, volume and induced anxiety and depression like behaviours in mice. Eur J Pharmacol.

[31] Sun L, Jia P, Zhang J, Zhang X, Zhang Y, Jiang H (2016). Production of inflammatory cytokines, cortisol, and Aβ1-40 in elderly oral cancer patients with postoperative delirium. Neuropsychiatr Dis Treat.

[32] Kronfol Z, Remick DG (2000). Cytokines and the brain: implications for clinical psychiatry. Am J Psychiatry.

[33] Huang Z, Meola D, John MP (2012). Dissecting the effects of endogenous brain IL-2 and normal versus autoreactive T lymphocytes on microglial responsiveness and T cell traffificking in response to axonal injury. Neurosci Lett.

[34] Bartoloni A, Polati E, Finco G, Facchin S, Rigo V, Gottin L (1995). The neuroendocrine and metabolic response to surgical stress. Chir Ital.

[35] Mark JS, Zaldy ST (2011). The role of inflammation in the pathogenesis of delirium and dementia in older adults: a review. CNS Neurosci Ther.

[36] Jiang CL, Lu CL (1998). Interleukin-2 and its effects in the central nervous system. Biol Signals Recept.

[37] Gozdzik W, Adamik B, Gozdzik A, Rachwalik M, Kustrzycki W, Kübler A (2014). Unchanged plasma levels of the soluble urokinase plasminogen activator receptor in elective coronary artery bypass graft surgery patients and cardiopulmonary bypass use. PLoS ONE.

[38] Paparella D, Yau TM, Young E (2002). Cardiopulmonary bypass induced inflammation: pathophysiology and treatment. An update. Eur J Cardiothorac Surg.

[39] Marco C, Maria RM, Sabrina B, Arturo C, Jan GJ (2018). Postoperative delirium and postoperative cognitive dysfunction: updates in pathophysiology, potential translational approaches to clinical practice and further research perspectives. Minerva Anestesiol.

[40] Anna C, Grazia MV, María-Jimena M-B, Federico N, Davide G, Mariarosa C (2019). Presepsin and procalcitonin levels as markers of adverse postoperative complications and mortality in cardiac surgery patients. Blood Purif.

[41] Stuart MG, Timothy DG, Jennifer LT, Ayumi KS, Alison WE, Wesley E (2011). Procalcitonin and C-reactive protein levels at admission as predictors of duration of acute brain dysfunction in critically ill patients. Crit Care.

